# Dimensional Accuracy of Regular- and Fast-Setting Vinyl Polysiloxane Impressions Using Customized Metal and Plastic Trays—An In Vitro Study

**DOI:** 10.3390/ma18092164

**Published:** 2025-05-07

**Authors:** Moritz Waldecker, Karla Jetter, Stefan Rues, Peter Rammelsberg, Andreas Zenthöfer

**Affiliations:** Department of Prosthetic Dentistry, University Hospital Heidelberg, University of Heidelberg, 69120 Heidelberg, Germany; karla.jetter@med.uni-heidelberg.de (K.J.); stefan.rues@med.uni-heidelberg.de (S.R.); peter.rammelsberg@med.uni-heidelberg.de (P.R.); andreas.zenthoefer@med.uni-heidelberg.de (A.Z.)

**Keywords:** accuracy, impression, fast-setting, regular-setting, vinyl polysiloxane, tray material

## Abstract

The aim of this study was to compare the dimensional accuracy of vinyl polysiloxane impressions differing in terms of curing time (regular-setting (RS) or fast-setting (FS)) in combination with different tray materials (metal (M) and plastic (P)). A typodont reference model simulated a partially edentulous maxilla. Reference points were given by center points of either precision balls welded to specific teeth or finishing-line centers of prepared teeth. These reference points enabled the detection of dimensional deviations between the digitized reference and the scans of the models achieved from the study impressions. Twenty impressions were made for each of the following four test groups: RS-M, RS-P, FS-M and FS-P. Global scan data accuracy was measured by distance and tooth axis deviations from the reference, while local accuracy was determined based on the trueness and precision of the abutment tooth surfaces. Statistical analysis was conducted using ANOVA accompanied by pairwise Tukey post hoc tests (α = 0.05). Most of the distances tended to be underestimated. Global accuracy was favorable; even for long distances, the mean absolute distance deviations were < 100 µm. Local accuracy was excellent for all test groups, with trueness ≤ 11 µm and precision ≤ 9 µm. Within the limitations of this study, all impression and tray materials were suitable to fabricate models with clinically acceptable accuracy.

## 1. Introduction

The prerequisite for accurately fitting dental restorations is a largely error-free transfer of the intraoral situation to the dental laboratory. Today, dentists have two options: conventional and digital impressions.

Digital impressions are currently the focus of scientific research and are increasingly being used in dental practices. Compared to conventional impressions, digital impressions are associated with increased patient comfort [1,2], and, depending on the indication, they can save time [1,2] and improve the accuracy of fit of fixed partial dentures (FPDs) [3,4]. However, there are limitations in certain clinical situations. A small mouth opening, highly subgingival preparation margins, poorly visible proximal contacts or anatomical features in the retromolar space can make digital impressions difficult or impossible to take. Due to these limitations, conventional impressions still play an important role in everyday dental practice.

Today, two main material groups are used to take impressions of teeth or implants: polyether and vinyl polysiloxane (VPS) [5,6]. Both materials have their advantages and disadvantages. As a result, there is no ideal impression material for every clinical situation [7,8], and the industry continues to improve existing impression materials or develop new ones.

In addition to the material properties, the chair time required to prepare and take an impression is an important factor for both dentists and patients. Patients prefer the impression method that is associated with the shortest chair time [1,2,9,10,11]. The comfort of the impression method seems to play a secondary role for patients [11]. For dentists, the efficiency of an impression method is particularly important [12]. It has been reported that small restorations can be fabricated faster with intraoral scanners than with conventional impressions [2,10,12,13]. To overcome these limitations and to offer an alternative to intraoral scanners, especially for small restorations, the dental industry has developed fast-setting impression materials in addition to regular-setting impression materials. As the composition differs from that of proven regular-setting impression materials, the proven suitability of the materials in terms of global and local accuracy is important.

Accuracy is also influenced by tray selection, the use of tray adhesives, and clinical parameters such as preparation design, saliva, bleeding from sulcus and the use of gingival retraction measures [14,15,16,17,18]. In terms of tray selection, the use of standard Rimlock metal trays is still very popular, as they enable reliable impressions to be taken [18,19,20]. For hygiene and sterilization purposes, disposable plastic trays are gaining in importance. Some studies have investigated Rimlock plastic trays and reported favorable results similar to those obtained with metal trays [18,21], while others have been unable to demonstrate comparability [22].

However, there have been no systematic studies on the global and local accuracy of fast-setting and regular-setting VPS materials in combination with Rimlock metal and plastic trays. The aim of this study was to compare the dimensional accuracy of two different VPS materials differing in terms of curing time (regular-setting and fast-setting) in combination with two different tray materials (metal and plastic) over short and long distances as well as their accuracy (trueness and precision) and angular deviations (tooth axes) at abutment tooth level. The null hypothesis was that accuracy would not be influenced by the impression material variant or tray material.

## 2. Materials and Methods

First, a dental model was virtually designed (computer-aided design (CAD)) which simulated the situation of a partially edentulous maxilla with central incisors, a right lateral incisor, canines, right first and second premolars, and first and second molars. The central incisors, canines and first molars were given full-crown preparations, simulating a patient treated with three crowns (right first molar, right canine and right central incisor) and a quadrant-spanning fixed partial denture (left central incisor–left canine–left first molar).

Second, precision balls were virtually added to this CAD file. For the virtual reference model, 5 precision balls (diameter = 3.175 mm) were placed occlusally to the second molars and the canines and between the central incisors. The five precision ball centers lay in a horizontal plane.

Third, the metallic model base, shaped like a maxilla, and the teeth were manufactured as separate parts (Colado^®^ CAD CoCr, Ivoclar Vivadent AG (Schaan, Liechtenstein)). Then, the surfaces of all the prepared teeth were digitized with a 25 µm measurement grid distance and high precision (µscan select+ with CLS4 sensor, Nano-Focus AG (Oberhausen, Germany); accuracy: 0.32 µm) to create a digital reference data set for each tooth.

**Figure 1:**
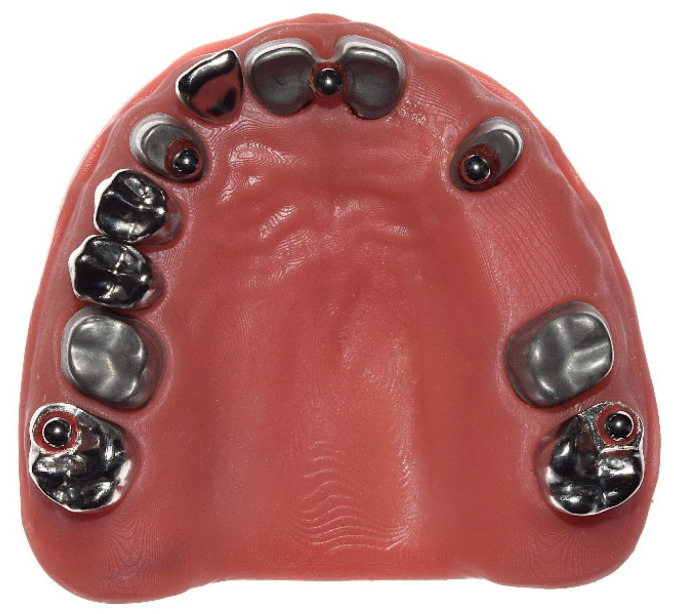
Occlusal view of the reference model.

Fourth, stainless-steel precision balls (diameter = 3.175 mm; grade 3) were welded on each of the teeth. The teeth were then welded onto the model base. The metallic model base was covered with a 3D-printed gingival mask (3D printer: Asiga MAX UV385, dentona AG (Dortmund, Germany); material: FREEPRINT^®^ gingiva, Detax GmbH & Co. KG (Ettlingen, Germany); Figure 1). After the welding process, measurements were made with a coordinate measuring machine (CMM; Mar-Vision 222, Hexagon Metrology, Wetzlar, Germany; accuracy: 1–2 µm) to determine the spatial positioning of the welded precision balls and all the prepared teeth.

**Figure 2:**
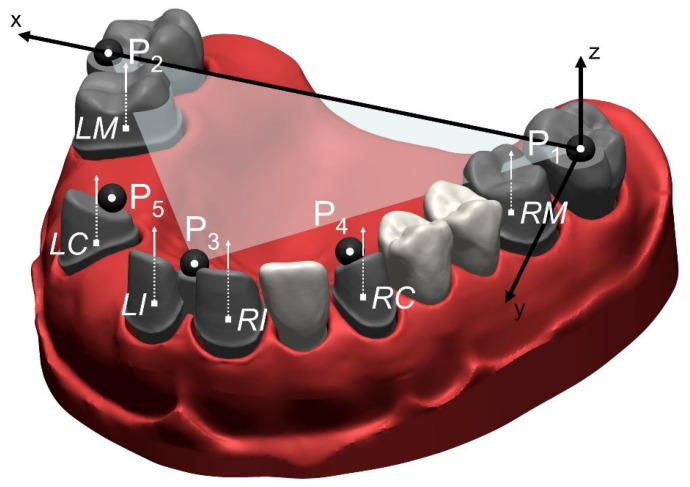
Three-dimensional view of the reference model with a global coordinate system, with P_1_ as the origin, the *x*-axis in the direction of P_1_P_2_, and the *xy*-plane defined by P_1_, P_2_ and P_3_. Center points of each precision ball (P_1_–P_5_) are marked with a circle. Center points of prepared teeth (RM = right molar, RC = right canine, RI = right incisor, LI = left incisor, LC = left canine, LM = left molar) are marked with a square. White arrows represent the tooth axis of the prepared teeth.

**Figure 3:**
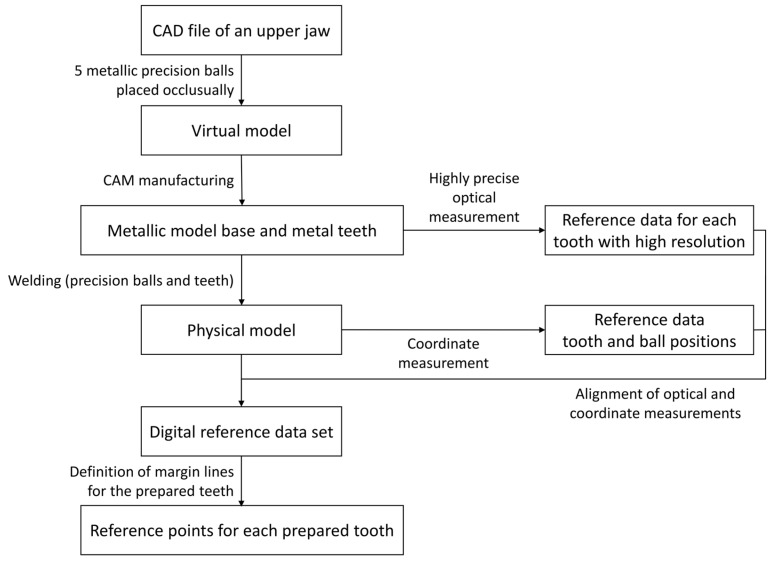
Flowchart of generating the reference data set.

Fifth, in order to obtain a digital reference data set, the previously digitized teeth were globally aligned with the coordinate measurement of the complete model (Geomagic Design X, 3D Systems (Rock Hill, SC, USA)). Spatial precision ball center positions were determined by minimizing the sum of all squared errors for any sphere with the given nominal radius and variable center position. For the reference data set, the five precision ball center points were numbered as shown in Figure 2, with P_1_ on the right second molar, P_2_ on the left second molar, P_3_ between the central incisors, P_4_ on the right canine and P_5_ on the left canine. A global coordinate system was defined as follows: P_1_ as the origin, the *x*-axis in the direction of P_1_P_2_, and the *xy*-plane defined by P_1_, P_2_ and P_3_, with the *y*-axis oriented in the anterior direction. For the definition of the center points at finishing-line level, the finishing lines were first defined as splines (Geomagic Design X, 3D Systems (Rock Hill, SC, USA)) on the high-resolution images of the prepared teeth. Then, the center points were gained by “extraction”, which resulted in the centers of mass of the respective lines. Tooth axes parallel to the *z*-axis of the global coordinate system were added at finishing-line center points. Thus, all tooth axes were parallel to each other in the reference situation, i.e., the angles between the corresponding axes amounted to 0 degrees. A flowchart of generating the reference data set can be seen in Figure 3.

**Figure 4:**
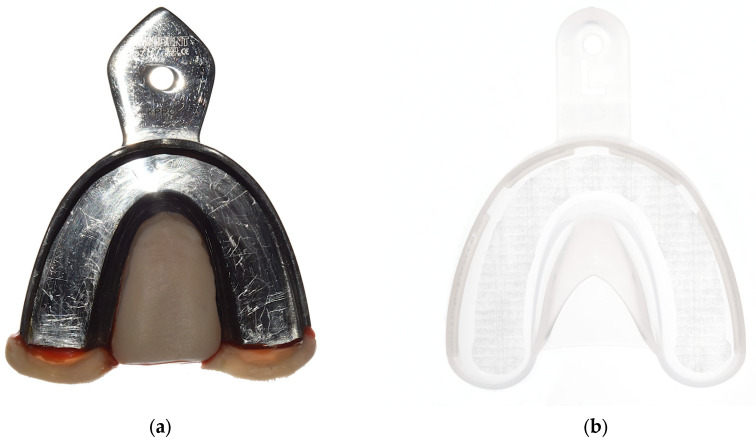
Used impression trays. (**a**) Customized metallic Rimlock tray. (**b**) Plastic tray.

**Figure 5:**
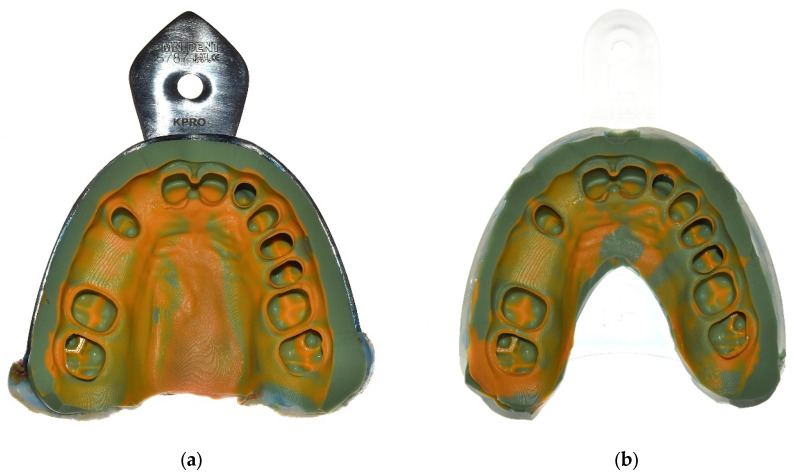
Vinyl polysiloxane impressions differing in terms of impression tray material. (**a**) Metallic Rimlock tray. (**b**) Plastic tray.

The model was inserted in a phantom head. All impressions were performed by the same experienced investigator (KJ). The impressions were performed using a dual-phase impression technique with VPS material in a regular- (Aquasil Ultra+ XLV and Aquasil Ultra+ Heavy Regular Set (RS), Dentsply Sirona (Charlotte, NC, USA)) and a fast-setting (Aquasil Ultra+ XLV and Aquasil Ultra+ Heavy Fast Set (FS), Dentsply Sirona (Charlotte, NC, USA)) variant at room temperature (23.9 ± 0.5 °C). Each impression material was used in combination with customized metallic Rimlock trays (M; impression tray (size: XL), Omnident Dental-Handelsgesellschaft mbH (Rodgau, Germany)) or plastic trays (P; impression tray (size: L), 3M GmbH (Seefeld, Germany)) (Figure 4 and Table 1). The metallic Rimlock trays were individualized with a palatal stop and a dorsal dam to ensure a permanent distance between the tray and the teeth and positional stability during the setting time and to support a seamless flow of the impression material to the tooth row (Figure 5). All impressions were removed from the model after 15 min (RS) or 7 min 30 s (FS), which is three times the clinical setting time for this VPS. In total, 80 impressions of the reference model were taken, equally distributed over the test groups (n = 20/group). Impressions were disinfected for 5 min (PrintoSept-ID, Alpro Medical GmbH (St. Georgen, Germany)) and then poured with type IV gypsum (esthetic-base gold, dentona AG (Dortmund, Germany)) no earlier than 30 min after disinfection. The saw-cut models were digitized using a laboratory scanner (D2000, 3shape A/S (Kopenhagen, Denmark)) with quality control software (Convince 2015, 3shape A/S (Kopenhagen, Denmark)) to generate a digital data set in STL file format.

The scans were aligned with the reference data set via the teeth in commercial CAD software (Geomagic Design X; 3D systems (Rock Hill, SC, USA)) by a best-fit algorithm. First, to determine the global scanning accuracy at precision ball level, scan surfaces were trimmed with predefined cutting surfaces such that only precision ball sections relevant for evaluation remained. Individual precision ball surfaces were exported to separate STL files. Afterwards, an automized evaluation (method of least squares; squared deviations at the triangle corner points were weighted with proportionate surface areas using MATLAB version R2022a, MathWorks (Natick, MA, USA)) was executed to determine the 5 precision ball centers (P1′–P5′, numbered in analogy to the reference model) by means of optimization and to calculate distance deviations between the scan and the reference situation. With 5 precision balls, there were 10 possible pairings. For better interpretation, these pairings were categorized (Table 2).

Second, to determine the global accuracy at tooth level, each reference tooth surface together with its local coordinate system was aligned separately to the scan data of the respective prepared tooth using a best-fit algorithm (Geomagic Design X, 3D Systems (Rock Hill, SC, USA)). For each two teeth, distances between the transformed coordinate origins (located at the center of the margin line) and angles between the transformed vertical axes were determined and compared to the values recorded for the reference model. With 6 abutment teeth, there were 15 possible pairings. For better interpretation, these pairings were categorized (Table 2). Since all reference distances were oriented (almost) horizontally, vertical deviations between each two reference points had a negligible impact on the calculated (total) distance deviation. Because of this, vertical distance deviations were reported separately.

The local accuracy of the individual surfaces of the prepared teeth within the margin line was analyzed in terms of trueness (mean mesh deviation between reference and scan) and precision (standard deviation of the mesh deviations along the surface). To evaluate trueness and precision, absolute (unsigned) values were used.

Besides the use of means and standard deviations (SDs) for descriptive result presentations, data were visualized using boxplot diagrams. Possible factors related to the dependent target variables were analyzed using ANOVA, and Tukey’s post hoc tests were performed for intergroup comparisons (SPSS 24, IBM (Armonk, NY, USA)).

## 3. Results

### 3.1. Global Accuracy

**Figure 6:**
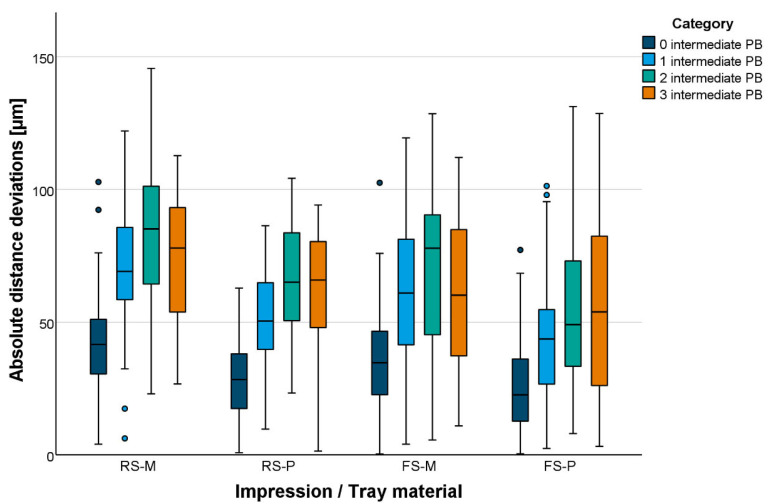
Boxplot diagram of the absolute distance deviations displayed for distances between precision ball centers.

**Figure 7:**
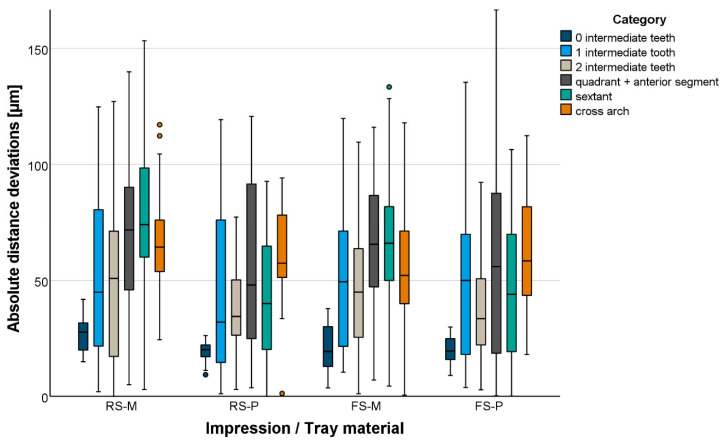
Boxplot diagram of the absolute distance deviations displayed for distances between abutment teeth measured at margin-line level.

Total distances were mainly underestimated (too short) independent of the test group. Absolute total distance deviations are displayed in Figure 6 and Figure 7. The global accuracy was favorable. Even for long distances, mean absolute distance deviations were <100 µm. Shorter distances were displayed more accurately than longer distances (Table 3). At precision ball level, the impression material setting variant had no effect in combination with plastic trays. In contrast, in combination with metal trays, fast-setting VPS led to smaller total distance deviations (*p* = 0.004). Plastic trays led to smaller total distance deviations when using fast- or regular-setting VPS (*p* < 0.001). At tooth level, the setting variant had no effect on the total distance deviations (*p* > 0.087). Total distance deviations between center points of margins were significantly smaller for plastic trays (*p* < 0.002).

**Figure 8:**
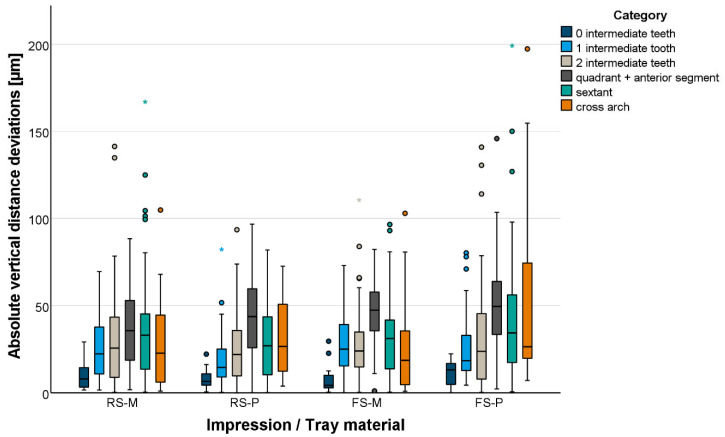
Boxplot diagram of the absolute vertical distance deviations displayed for distances between abutment teeth measured at margin-line level.

Vertical distance deviations are displayed in Figure 8. When using metal trays, no significant difference was found between the setting variants. With plastic trays, regular-setting VPS (RS-P) provided significantly fewer vertical distance deviations (*p* < 0.001) compared to fast-setting VPS (FS-P). No significant difference could be observed between metal (RS-M) and plastic trays (RS-P) when using regular-setting VPS. Using fast-setting VPS, vertical distance deviations were significantly smaller with metal trays (*p* = 0.034).

The mean angular deviations between the tooth axes (*z*-axes) ranged between 0.10° and 0.57° for all test groups (Table 4). Maximum vertical axis deviations ranged between 0.14° and 1.31°. The setting variant had a significant effect when using plastic trays. With regular-setting VPS, tooth axis deviations were significantly smaller (*p* = 0.006). With metal trays, tooth axis deviations were significantly smaller independent of the used setting-variant (*p* < 0.012).

### 3.2. Local Accuracy

**Figure 9:**
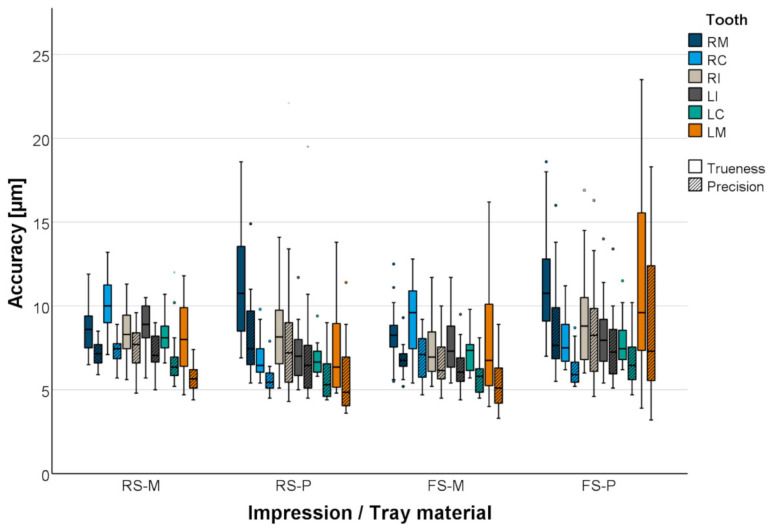
Boxplot diagram of the accuracy (trueness and precision) of prepared teeth.

The local accuracy of the gypsum master casts was comparable in all test groups. Excellent accuracy was obtained for all abutment teeth (mean trueness ≤ 11 µm, mean precision ≤ 9 µm; Figure 9 and Table 5). Setting variant and tray material had a significant influence on local accuracy. The trueness of the prepared teeth was smaller for regular-setting VPS impressions with plastic trays (RS-P) compared to metal trays (RS-M; *p* = 0.031). In contrast, the trueness of the prepared teeth was larger for fast-setting VPS impressions with plastic trays (FS-P) compared to metal trays (FS-M; *p* < 0.001). Using metal trays in combination with fast-setting VPS (FS-M) led to more accurate abutment teeth (*p* = 0.030) compared to regular-setting VPS (RS-M). Using plastic trays, the combination with regular-setting VPS was favorable (RS-P; *p* < 0.001) compared to fast-setting VPS (FS-P).

## 4. Discussion

The null hypothesis that accuracy would not be influenced by the impression material variant or the tray material had to be rejected. With respect to global and local accuracy, the differences between the test groups were small but in some cases statistically significant.

In general, the setting process of an impression material generates heat, and the contraction of the impression material exerts a certain amount of force on the tray. In preliminary tests on temperature and force development, it was shown that the temperature of fast-setting VPS was initially warmer than the temperature of regular-setting VPS immediately after mixing. The absolute temperature increase during the setting process was comparable for both setting variants but was much faster with fast-setting VPS.

In this study, the use of plastic trays led to significantly smaller total distance deviations independent of the used setting variant. This result is partly contradictory to findings in the literature. A study on the influence of tray rigidity and impression technique on the accuracy of impressions with VPS showed that plastic trays produced less accurate impressions than metal trays [22]. In a study by Rues et al. on the influence of disposable plastic trays on the impression accuracy of polyether and VPS, different results were found depending on the measuring level. The evaluation strategy in this study was similar to that in the present study. While distance deviations at precision ball level were significantly smaller when using plastic trays compared with metal trays, the deviations with plastic trays were significantly greater at margin level [18]. The reasons for the differences between the studies can only be speculated on. The shape, material composition and additional retention elements of the plastic trays could have had an influence on accuracy.

Vertical distance deviations were significantly larger with fast-setting VPS in combination with plastic trays compared to the other test groups. At least for longer distances (larger than one quadrant), the greater heat and force development of fast-setting VPS seems to be advantageous for vertical distance deviations when using metal trays.

With regard to the local accuracy of the impressions/plaster casts, both trueness and precision were excellent, independent of the impression material variant and the tray material. The local accuracy of the prepared teeth was within the range of values found in previous studies [18,19,20]. The accuracy differed only slightly between the test groups, although it was statistically significant. However, both the impression materials and the tray materials are suitable for clinical use. The in vitro results show that highly accurate impressions of single crowns, fixed partial dentures and even a complete dental arch are possible with all the test groups.

Clinically acceptable impressions could be made with both setting variants and both tray materials. The accuracy differed only slightly between the test groups. The decision as to which of the two setting variants or which trays to use will not primarily depend on the achievable accuracy in everyday practice. The number of abutment teeth or implants is particularly limiting for the fast-setting VPS. With an intraoral processing time of 35 sec, compared to 1 min 10 sec for regular-setting VPS, there are time limits on the material side.

This study has several limitations. It was an in vitro investigation focused on a specific partially edentulous scenario involving both prepared and unprepared metal teeth, which means the findings may not be applicable to other dental situations. Future research should explore additional models representing various partially edentulous situations. Nevertheless, it is important to note that metal teeth do not exhibit significantly higher demolding forces compared to natural teeth. The material properties of the metal reference teeth regarding demolding forces were comparable to those of natural teeth [18]. A further limitation is the fact that the workflow used in this study represents a best-case scenario. The impression trays were fixed and completely immobile during the setting of the impression material, which means that clinically relevant influences of patient movements or changes in the position of the impression tray affecting accuracy were not observed. In addition, it was not possible to investigate the effects of moisture or the presence of saliva, sulcular fluid and blood in vitro.

## 5. Conclusions

Within the limitations of this study, the following conclusions were drawn:

1. RS-VPS and FS-VPS have adequate accuracy in vitro, which suggests a successful clinical application.
2. The use of metallic trays or plastic trays leads to adequate accuracy in vitro, which suggests a successful clinical application.
3. Short distances are displayed more accurately than long distances regardless of material variant or tray material.

## Figures and Tables

**Table 1:** Test groups differing in terms of impression material and tray material.

Test Group	Impression Material	Tray Material
RS-M	Aquasil Ultra+ Heavy Regular Set/Aquasil Ultra+ XLV Regular Set	Metal
RS-P		Plastic
FS-M	Aquasil Ultra+ Heavy Fast Set/Aquasil Ultra+ XLV Fast Set	Metal
FS-P		Plastic

RS-M, Regular Set–Metal Tray; RS-P, Regular Set–Plastic Tray; FS-M, Fast Set–Metal Tray; FS-P, Fast Set–Plastic Tray.

**Table 2:** Distance categories and corresponding distances defined by center points at the margin level of the prepared teeth or by center points of precision balls.

Level	Distance Category	Distance
Precision ball	No intermediate precision ball	P_1_–P_4_, P_2_–P_5_, P_3_–P_4_, P_3_–P_5_
	1 intermediate precision ball	P_1_–P_3_, P_2_–P_3_, P_4_–P_5_
	2 intermediate precision balls	P_1_–P_5_, P_2_–P_4_
	3 intermediate precision balls	P_1_–P_2_
Prepared teeth	No intermediate tooth	RI-LI
	1 intermediate tooth	RI-RC, LI-LC
	2 intermediate teeth	RC-RM, LC-LM, RC-LI, RI-LC
	Quadrant + anterior segment	RI-RM, LI-LM, RC-LC
	Sextant	RM-LC, RC-LM
	Cross-arch	RM-LM

P_1_–P_5_, Precision Ball Centers 1–5; RM, Right Molar; RC, Right Canine; RI, Right Incisor; LI, Left Incisor; LC, Left Canine; LM, Left Molar.

**Table 3:** Distance deviations between center points of precision balls and of margins.

Test Group	Level	Distance	Distance Deviations [µm]				
			Mean Value	Standard Deviation	Minimum	Median	Maximum
RS-M	Precision ball	P_1_–P_2_	75	25	27	78	113
		P_1_–P_3_	72	21	33	74	110
		P_1_–P_4_	42	15	8	42	76
		P_1_–P_5_	77	29	23	83	146
		P_2_–P_3_	80	22	32	79	122
		P_2_–P_4_	89	26	46	93	134
		P_2_–P_5_	52	21	12	47	103
		P_3_–P_4_	40	19	4	42	74
		P_3_–P_5_	33	13	6	30	57
		P_4_–P_5_	60	23	6	61	103
	Prepared teeth	RM-RC	16	14	0	12	60
		RM-RI	81	29	27	79	140
		RM-LI	55	28	6	59	123
		RM-LC	78	32	14	73	146
		RM-LM	68	24	24	64	117
		RC-RI	72	26	22	80	125
		RC-LI	65	29	9	71	127
		RC-LC	81	36	22	75	150
		RC-LM	79	36	3	74	153
		RI-LI	27	8	15	28	42
		RI-LC	64	26	9	68	108
		RI-LM	104	35	16	103	176
		LI-LC	27	16	2	22	55
		LI-LM	58	30	5	52	113
		LC-LM	48	24	0	49	94
RS-P	Precision ball	P_1_–P_2_	60	27	1	66	94
		P_1_–P_3_	43	17	10	44	72
		P_1_–P_4_	20	13	1	22	46
		P_1_–P_5_	59	22	23	62	104
		P_2_–P_3_	54	15	29	53	78
		P_2_–P_4_	71	21	30	70	100
		P_2_–P_5_	35	14	13	37	63
		P_3_–P_4_	31	12	1	31	45
		P_3_–P_5_	25	13	4	25	47
		P_4_–P_5_	55	18	10	56	86
	Prepared teeth	RM-RC	29	19	4	28	76
		RM-RI	85	24	10	92	121
		RM-LI	54	16	19	58	82
		RM-LC	68	17	24	71	93
		RM-LM	60	22	1	57	94
		RC-RI	72	21	17	76	119
		RC-LI	47	16	3	49	77
		RC-LC	33	20	1	30	69
		RC-LM	23	18	0	23	69
		RI-LI	19	5	9	20	26
		RI-LC	43	15	10	44	71
		RI-LM	76	15	42	77	101
		LI-LC	16	10	1	15	33
		LI-LM	26	14	4	29	50
		LC-LM	28	13	4	30	57
FS-M	Precision ball	P_1_–P_2_	60	29	11	60	112
		P_1_–P_3_	65	32	4	66	118
		P_1_–P_4_	33	25	0	36	76
		P_1_–P_5_	69	29	20	69	129
		P_2_–P_3_	64	28	5	64	119
		P_2_–P_4_	72	28	6	82	107
		P_2_–P_5_	44	23	5	40	103
		P_3_–P_4_	39	14	7	37	73
		P_3_–P_5_	29	9	14	27	47
		P_4_–P_5_	54	20	10	58	89
	Prepared teeth	RM-RC	21	18	1	16	68
		RM-RI	75	21	32	74	116
		RM-LI	55	20	8	51	92
		RM-LC	68	18	23	67	99
		RM-LM	57	28	1	52	118
		RC-RI	69	20	31	70	120
		RC-LI	61	23	15	58	110
		RC-LC	71	27	19	68	122
		RC-LM	64	35	4	62	133
		RI-LI	21	10	4	19	38
		RI-LC	59	17	31	64	95
		RI-LM	92	20	59	92	133
		LI-LC	29	18	10	22	77
		LI-LM	55	28	7	49	110
		LC-LM	38	22	3	38	91
FS-P	Precision ball	P_1_–P_2_	54	34	3	54	129
		P_1_–P_3_	44	28	3	44	98
		P_1_–P_4_	25	20	2	18	77
		P_1_–P_5_	54	34	8	45	131
		P_2_–P_3_	43	23	2	40	101
		P_2_–P_4_	57	26	20	55	121
		P_2_–P_5_	29	18	1	27	68
		P_3_–P_4_	32	18	4	33	67
		P_3_–P_5_	18	14	0	19	49
		P_4_–P_5_	46	23	20	46	95
	Prepared teeth	RM-RC	29	22	3	24	92
		RM-RI	87	29	51	79	169
		RM-LI	55	22	18	56	95
		RM-LC	73	20	33	72	106
		RM-LM	62	27	18	58	112
		RC-RI	74	26	42	70	135
		RC-LI	50	15	25	52	75
		RC-LC	35	26	7	31	104
		RC-LM	35	28	0	32	105
		RI-LI	20	5	9	20	30
		RI-LC	35	20	6	31	79
		RI-LM	66	17	23	64	106
		LI-LC	25	19	4	18	61
		LI-LM	22	20	0	19	88
		LC-LM	31	14	7	30	59

RS-M, Regular Set–Metal Tray; RS-P, Regular Set–Plastic Tray; FS-M, Fast Set–Metal Tray; FS-P, Fast Set–Plastic Tray; P_1_–P_5_, Precision Ball Centers 1–5; RM, Right Molar; RC, Right Canine; RI, Right Incisor; LI, Left Incisor; LC, Left Canine; LM, Left Molar.

**Table 4:** Vertical angular deviations for prepared teeth.

Test Group	Category	Angular Deviations [°]				
		Mean Value	Standard Deviation	Minimum	Median	Maximum
RS-M	0 intermediate teeth	0.10	0.07	0.02	0.10	0.27
	1 intermediate tooth	0.17	0.11	0.01	0.16	0.43
	2 intermediate teeth	0.18	0.10	0.00	0.14	0.50
	Quadrant + anterior segment	0.14	0.08	0.03	0.14	0.38
	Sextant	0.32	0.08	0.15	0.31	0.53
	Cross-arch	0.21	0.09	0.04	0.19	0.39
RS-P	0 intermediate teeth	0.10	0.03	0.04	0.09	0.14
	1 intermediate tooth	0.16	0.12	0.00	0.13	0.50
	2 intermediate teeth	0.17	0.09	0.01	0.15	0.45
	Quadrant + anterior segment	0.24	0.14	0.00	0.24	0.66
	Sextant	0.33	0.15	0.01	0.32	0.61
	Cross-arch	0.47	0.20	0.07	0.46	0.78
FS-M	0 intermediate teeth	0.08	0.04	0.03	0.08	0.16
	1 intermediate tooth	0.17	0.10	0.02	0.15	0.49
	2 intermediate teeth	0.13	0.10	0.02	0.10	0.51
	Quadrant + anterior segment	0.16	0.09	0.02	0.14	0.38
	Sextant	0.25	0.09	0.06	0.25	0.40
	Cross-arch	0.23	0.09	0.08	0.24	0.38
FS-P	0 intermediate teeth	0.09	0.03	0.04	0.08	0.15
	1 intermediate tooth	0.18	0.14	0.01	0.14	0.58
	2 intermediate teeth	0.19	0.11	0.02	0.16	0.57
	Quadrant + anterior segment	0.30	0.17	0.07	0.26	0.74
	Sextant	0.39	0.25	0.06	0.33	1.18
	Cross-arch	0.57	0.31	0.07	0.55	1.31

RS-M, Regular Set–Metal Tray; RS-P, Regular Set–Plastic Tray; FS-M, Fast Set–Metal Tray; FS-P, Fast Set–Plastic Tray.

**Table 5:** Trueness (precision) for individual prepared teeth.

Test Group	Prepared Tooth	Accuracy [µm]				
		Mean Value	Standard Deviation	Minimum	Median	Maximum
RS-M	RM	9 (7)	1 (1)	7 (6)	9 (7)	12 (9)
	RC	10 (7)	2 (1)	7 (6)	10 (7)	13 (9)
	RI	8 (8)	1 (1)	6 (5)	8 (8)	11 (10)
	LI	9 (7)	1 (1)	6 (5)	9 (7)	11 (9)
	LC	8 (7)	1 (2)	7 (5)	8 (6)	11 (12)
	LM	8 (6)	2 (1)	5 (4)	8 (6)	12 (7)
RS-P	RM	11 (8)	3 (2)	7 (5)	11 (7)	19 (15)
	RC	7 (6)	1 (1)	5 (5)	6 (5)	10 (8)
	RI	8 (8)	2 (4)	5 (4)	8 (7)	14 (22)
	LI	7 (7)	2 (3)	5 (5)	7 (6)	12 (20)
	LC	7 (6)	1 (1)	6 (4)	7 (5)	9 (9)
	LM	7 (6)	3 (2)	5 (4)	6 (5)	14 (11)
FS-M	RM	8 (7)	2 (1)	6 (5)	8 (7)	13 (9)
	RC	9 (7)	2 (1)	5 (5)	10 (7)	13 (9)
	RI	7 (7)	2 (1)	5 (5)	7 (6)	12 (10)
	LI	8 (6)	2 (1)	5 (4)	7 (6)	12 (10)
	LC	7 (6)	1 (1)	6 (5)	7 (6)	10 (8)
	LM	8 (5)	3 (2)	4 (3)	7 (5)	16 (9)
FS-P	RM	11 (9)	3 (3)	7 (6)	11 (8)	19 (16)
	RC	8 (6)	2 (1)	6 (5)	8 (6)	11 (9)
	RI	9 (8)	3 (3)	6 (5)	9 (8)	17 (16)
	LI	8 (8)	2 (2)	5 (5)	8 (7)	14 (13)
	LC	8 (7)	1 (1)	6 (5)	7 (6)	12 (10)
	LM	11 (9)	6 (5)	4 (3)	10 (7)	24 (18)

RS-M, Regular Set–Metal Tray; RS-P, Regular Set–Plastic Tray; FS-M, Fast Set–Metal Tray; FS-P, Fast Set–Plastic Tray; RM, Right Molar; RC, Right Canine; RI, Right Incisor; LI, Left Incisor; LC, Left Canine; LM, Left Molar.

## Data Availability

The original contributions presented in the study are included in the article, further inquiries can be directed to the corresponding author.
